# Localized Nasal Dorsum Skin Necrosis: A Rare Complication After Rhinoplasty

**DOI:** 10.1002/ccr3.71416

**Published:** 2025-11-05

**Authors:** Abdullah D. Alotaibi

**Affiliations:** ^1^ College of Medicine, University of Hail Hail Saudi Arabia

**Keywords:** external splint, nasal dorsum, pressure injury, rhinoplasty, skin necrosis

## Abstract

Nasal dorsum skin necrosis is a rare but serious complication of rhinoplasty, with an incidence of < 1%. This case report presents a healthy young female who developed localized skin necrosis following primary aesthetic rhinoplasty, likely due to prolonged pressure from an external aluminum nasal splint and delayed postoperative follow‐up. The lesion was successfully managed conservatively using fusidic acid ointment and silicone gel, resulting in complete epithelialization without the need for surgical intervention. This case highlights the critical importance of appropriate splint material selection, timely postoperative evaluation, patient compliance, and early initiation of conservative therapy to prevent and manage such complications.

**Summary**

- Nasal dorsum skin necrosis after rhinoplasty is rare but preventable.
- Timely follow‐up, appropriate splint selection, and early intervention are essential to avoid serious aesthetic complications.
- Even delayed cases can be managed conservatively with favorable outcomes if recognized and treated appropriately.

## 1 Introduction

Rhinoplasty continues to be one of the most commonly performed cosmetic surgeries worldwide, consistently ranking among the top five aesthetic procedures. As reported in the 2022 Global Survey by the International Society of Aesthetic Plastic Surgery (ISAPS), there has been a notable 19.3% increase in the number of cosmetic procedures performed by plastic surgeons compared to previous years [1]. In 2020 alone, an estimated 850,000 rhinoplasty surgeries were carried out globally. The procedure is commonly indicated for correcting congenital or acquired nasal deformities, posttraumatic structural abnormalities, and functional issues such as impaired nasal breathing. Despite ongoing improvements in surgical methods, instruments, and postoperative protocols, rhinoplasty remains associated with a risk of complications.

The most frequently encountered postoperative complications include periorbital oedema, ecchymosis, infection, septal deviation, nasal obstruction, and dissatisfaction with cosmetic outcomes [2, 3]. Skin necrosis on the nasal dorsum after rhinoplasty is exceptionally rare, reported in < 1% of cases. For instance, in a case series of 244 rhinoplasties performed by a single surgeon, two patients developed dorsal skin necrosis, yielding an incidence rate of approximately 0.8% [4]. This form of necrosis is typically attributed to compromised blood supply, which may result from excessive intraoperative trauma, overly tight dressings, or prolonged pressure from rigid external nasal splints [5].

Anatomically, the nasal dorsum is covered by thin skin with relatively limited subdermal vascularization, making it particularly vulnerable to ischemic injury under sustained compression [6]. Although rare, nasal skin necrosis can lead to aesthetically disfiguring outcomes and significant psychological distress, particularly in younger patients undergoing cosmetic procedures.

Delayed recognition of early ischemic changes, often due to postponed or missed postoperative follow‐up, may further exacerbate tissue damage and complicate recovery. The present case illustrates the importance of vigilant postoperative monitoring, appropriate splint material selection, and early intervention to prevent and manage this uncommon but impactful complication.

## 2 Case History/Examination

A 20‐year‐old healthy, nonsmoking female underwent open rhinoplasty for dorsal hump reduction and nasal septal deviation correction. The procedure included marginal and transcolumellar incisions, dorsal rasping, placement of a columellar strut graft, and bilateral lateral osteotomies. The surgery was completed without any intraoperative complications.

**FIGURE 1:**
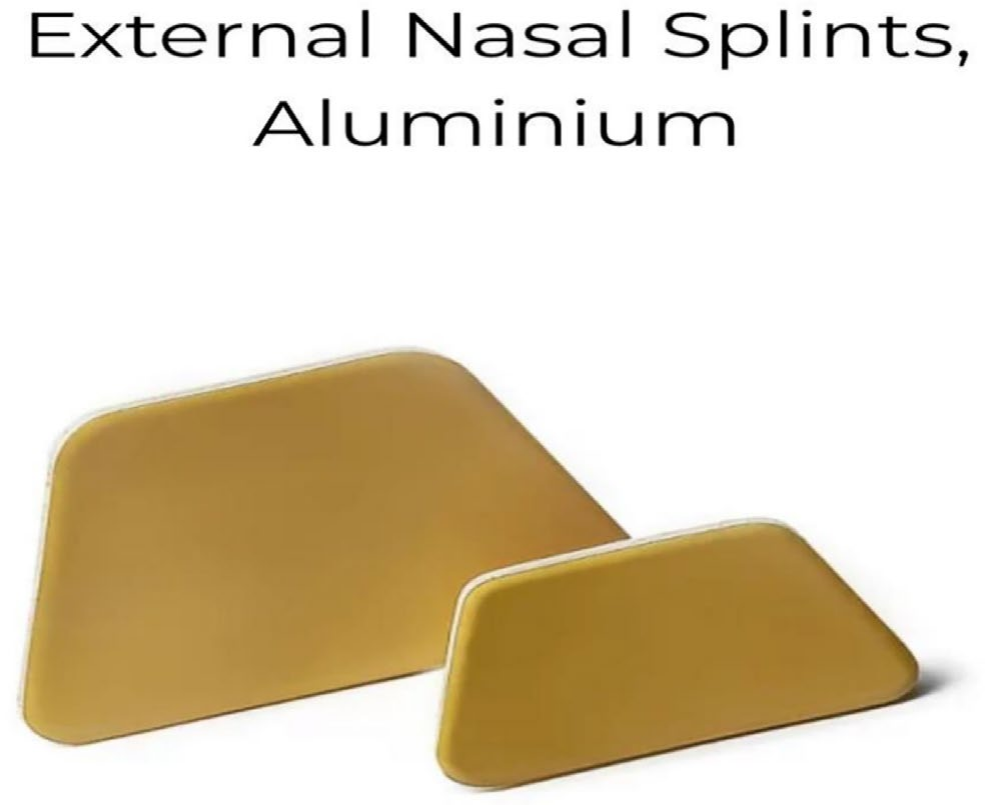
External aluminum nasal splint used postoperatively.

Postoperatively, the nasal bones were supported using a rigid, preformed aluminum external nasal splint (Figure 1) secured with adhesive tape.

## 3 Differential Diagnosis/Investigation and Treatment

Upon delayed presentation, 20 days after surgery, removal of the nasal splint revealed a well‐demarcated necrotic ulcer (1 × 0.25 cm) over the mid‐nasal dorsum. The ulcer exhibited slough and mild erythema, with no systemic signs of infection, cellulitis, or hypersensitivity.

**Differential diagnosis included:**

Pressure‐induced ischemic necrosis.

Contact dermatitis or hypersensitivity reaction.

Infection (bacterial/fungal).

Autoimmune‐related vasculitis.

Based on the history and clinical findings, pressure necrosis was deemed the most likely diagnosis.

**Treatment:**

Gentle saline cleansing of the wound.

Twice‐daily topical fusidic acid ointment.

Strict photoprotection.

After healing, silicone gel was used to minimize scarring.

## 4 Outcome and Follow‐Up

The patient unfortunately missed her follow‐up appointment despite being advised about wound care and the importance of follow‐up. At 10 months, she returned, and the area had fully healed with a flat, faint scar and no functional or aesthetic deformity. During that period she reported continuing the wound care and photoprotection measures. Surgical intervention was not required.

## 5 Conclusion and Results (Outcome and Follow‐Up)

In this case, delayed follow‐up following open rhinoplasty led to localized ischemic necrosis of the nasal dorsum, attributed to prolonged application of a rigid aluminum splint. Despite the late presentation, the lesion remained superficial and was effectively managed through conservative measures, including wound hygiene, topical antibiotic therapy, silicone‐based scar management, and photoprotection.

**FIGURE 2:**
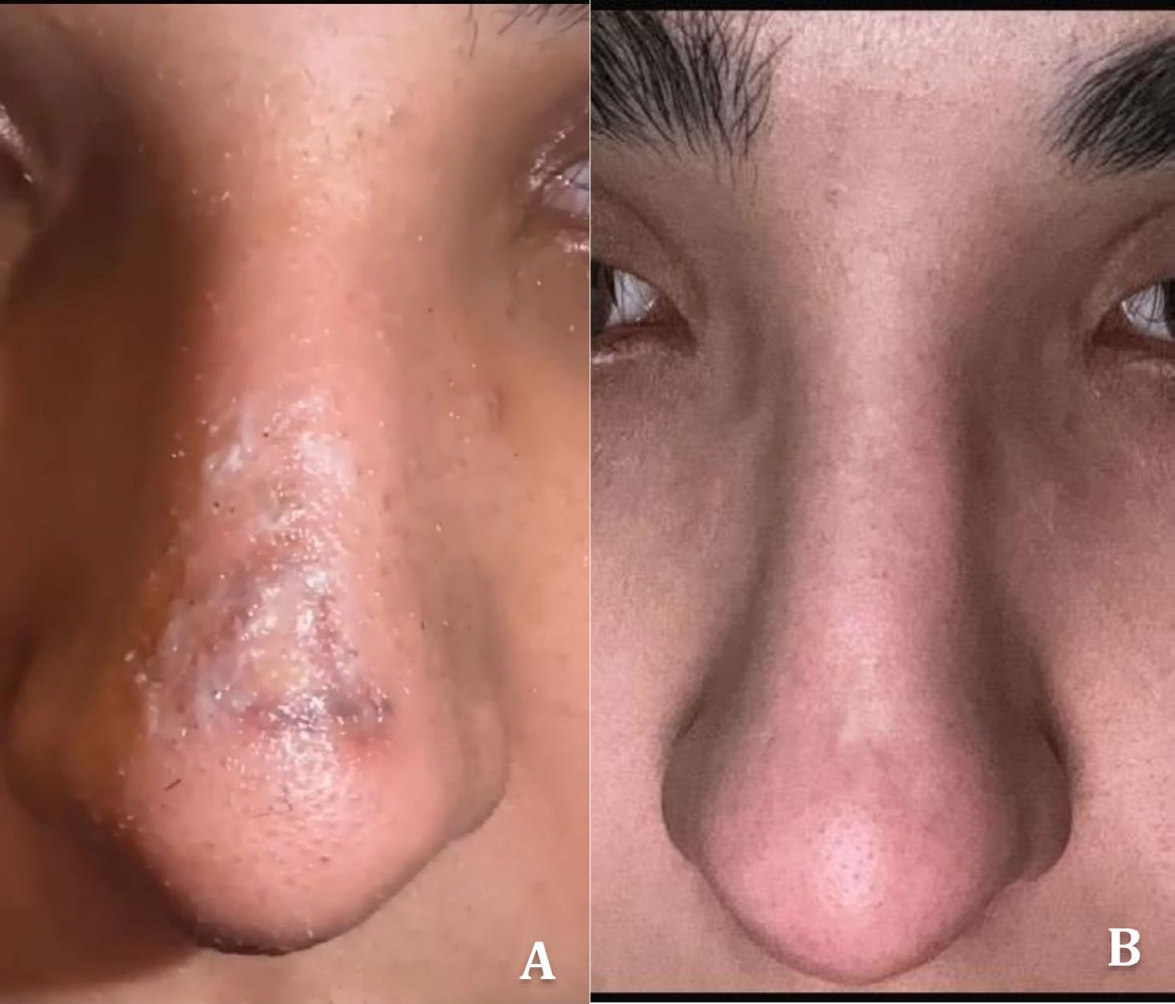
(A) Clinical appearance at 20 days following surgery showing skin necrosis over the nasal dorsum. (B) Ten‐month follow‐up showing complete healing with minimal scarring.

At the 10‐month follow‐up, the patient exhibited complete healing with a flat, hypopigmented scar and no contour deformity or functional impairment. Surgical revision was not required, emphasizing that superficial skin necrosis, when recognized and managed appropriately, even in delayed cases, can have favorable outcomes without invasive intervention (Figure 2).

This case highlights the importance of strict adherence to postoperative follow‐up schedules, early identification of vascular compromise, and patient education regarding warning signs. Furthermore, it emphasizes the role of splint material selection in reducing the risk of ischemic complications, particularly in patients with thinner nasal skin or prolonged splint application. Prevention remains the cornerstone, but timely conservative therapy can yield excellent results in selected cases.

## 6 Discussion

### 6.1 Etiology and Pathophysiology

The underlying pathophysiology is primarily ischemic. Sustained external pressure from rigid nasal splints can compromise capillary blood flow, particularly in the setting of surgically traumatized tissue. The nasal dorsum is especially susceptible due to its thin skin and limited subdermal vascular network. This vulnerability is exacerbated after the resolution of postoperative edema, which may allow splints to exert greater pressure directly on the overlying skin [7].

### 6.2 Risk Factors and Patient Vulnerability

A variety of patient‐specific and procedural factors may predispose individuals to skin necrosis following rhinoplasty. These include the following:

- Prolonged application of rigid external nasal splints can exert continuous pressure on the skin and compromise local perfusion [8].
- Delayed postoperative follow‐up, as seen in the present case, leads to late identification of early ischemic changes.
- Thin or overly dissected nasal skin, which may have reduced vascularity and resilience to external stress [5].
- Preoperative use of topical retinoids or corticosteroids can impair skin integrity and wound healing [4].
- Allergic or irritant contact reactions to adhesives or splint materials, contribute to localized inflammation and vascular compromise [7].
- Smoking or systemic conditions affecting microvascular circulation, such as diabetes mellitus or connective tissue disorders, which are known to impair wound healing [9].

### 6.3 Splint Design and Material Considerations

Aluminum nasal splints, though effective for stabilizing nasal bones postrhinoplasty, are rigid and nonconforming, which can lead to pressure‐induced ischemia and skin necrosis if applied too tightly or worn for extended periods. In contrast, thermoplastic or padded splints better accommodate postoperative edema and distribute pressure more evenly. Studies have shown that using padded or thermoplastic splints significantly reduces the risk of dorsal skin complications compared to rigid aluminum types [10].

### 6.4 Importance of Timely Follow‐Up

Close postoperative monitoring during the initial 7–10 days is critical for early identification of complications such as vascular compromise or skin breakdown. In the present case, delayed follow‐up allowed evolving ischemic injury to progress unnoticed. Early signs, including skin discoloration, localized tenderness, or crusting, should prompt immediate clinical evaluation and, if necessary, splint removal to prevent irreversible damage [11, 12].

Patient education is equally important. Patients should be thoroughly counseled on the signs of splint‐related complications and instructed to seek prompt medical attention if such symptoms occur.

### 6.5 Management Strategies

Superficial nasal skin necrosis without exposure of deeper tissues can often be managed conservatively. Key principles of management include the following:

- Early removal of the causative pressure source.
- Topical antibiotics (e.g., fusidic acid) to prevent secondary bacterial colonization or infection [13].
- Silicone‐based gels to reduce scar formation during the healing process [14].
- Photoprotection, especially during the remodeling phase, to minimize postinflammatory hyperpigmentation and optimize aesthetic outcomes.

In cases where nasal dorsum necrosis is extensive, involves deeper dermal or subcutaneous layers, or does not respond to conservative treatment, surgical intervention may be necessary. Management options include local wound debridement, full‐thickness skin grafting, or regional flap reconstruction, which are selected based on the defect size, depth, and location [15]. These techniques aim to restore skin integrity and preserve nasal contour. In addition to traditional surgical methods, regenerative therapies such as autologous fat grafting and platelet‐rich plasma (PRP) have gained popularity for their ability to enhance tissue regeneration and reduce scarring. Fat grafts serve not only as volume fillers but also promote dermal repair through adipose‐derived stem cells and growth factors. When combined with PRP, which contains high concentrations of platelets and cytokines, the graft survival rate and regenerative effect may be significantly improved [16, 17]. Clinical reports and small case series have documented successful outcomes using fat grafting with or without PRP in cases of posttraumatic or postsurgical nasal scarring, including necrosis following rhinoplasty. However, while promising, these techniques require further validation in large‐scale controlled studies. It should be noted that fat grafting and PRP are not suitable for all cases. They should be avoided when there is active infection or unstable wound surfaces as these factors can affect healing and graft survival.

### 6.6 Prognosis

When recognized early and managed appropriately, superficial nasal dorsum necrosis typically heals with favorable outcomes and minimal long‐term sequelae [2, 18]. In contrast, delayed diagnosis or insufficient intervention may result in adverse cosmetic consequences such as hypertrophic scarring, skin contracture, or nasal contour deformities, potentially requiring secondary reconstructive procedures. In the present case, although initial recognition was delayed, subsequent conservative management, including topical wound care and close monitoring, led to complete resolution without the need for surgical revision, emphasizing the potential efficacy of nonsurgical strategies when instituted appropriately.

## Author Contributions

**Abdullah D. Alotaibi:** conceptualization, investigation, methodology, resources, validation, writing – original draft, writing – review and editing.

## Ethics Statement

In compliance with the Helsinki Declaration, written and informed consent was obtained from the patient before proceeding.

## Consent

The author reviewed this manuscript and agreed to submit it. Informed consent for publication was obtained from the patient in accordance with the journal's policy.

## Conflicts of Interest

The author declares no conflicts of interest.

## Data Availability

The data that support the findings of this study are available from the corresponding author upon reasonable request.
